# Complement C4, Infections, and Autoimmune Diseases

**DOI:** 10.3389/fimmu.2021.694928

**Published:** 2021-07-14

**Authors:** Hongbin Wang, Mengyao Liu

**Affiliations:** ^1^ Master Program of Pharmaceutical Sciences College of Graduate Studies, California Northstate University, Elk Grove, CA, United States; ^2^ Department of Pharmaceutical and Biomedical Sciences College of Pharmacy, California Northstate University, Elk Grove, CA, United States; ^3^ Department of Basic Science College of Medicine, California Northstate University, Elk Grove, CA, United States

**Keywords:** complement, C4, C4a, C4d, infections, autoimmune diseases

## Abstract

Complement C4, a key molecule in the complement system that is one of chief constituents of innate immunity for immediate recognition and elimination of invading microbes, plays an essential role for the functions of both classical (CP) and lectin (LP) complement pathways. Complement C4 is the most polymorphic protein in complement system. A plethora of research data demonstrated that individuals with C4 deficiency are prone to microbial infections and autoimmune disorders. In this review, we will discuss the diversity of complement C4 proteins and its genetic structures. In addition, the current development of the regulation of complement C4 activation and its activation derivatives will be reviewed. Moreover, the review will provide the updates on the molecule interactions of complement C4 under the circumstances of bacterial and viral infections, as well as autoimmune diseases. Lastly, more evidence will be presented to support the paradigm that links microbial infections and autoimmune disorders under the condition of the deficiency of complement C4. We provide such an updated overview that would shed light on current research of complement C4. The newly identified targets of molecular interaction will not only lead to novel hypotheses on the study of complement C4 but also assist to propose new strategies for targeting microbial infections, as well as autoimmune disorders.

## Introduction

Complement system plays a pivotal role in human innate immunity defending microbial infections, eliminating foreign pathogens, and maintaining tissue homeostasis. The activation of the complement system induces the increased production of cytokines, chemokines, and other innate defense molecules. In addition, complement activation fragments (*e.g.*, anaphylatoxin C3a and C5a) significantly increased the recognition of antigens by follicular dendritic cells and B cells and induced the humoral adaptive immune response and production of antibodies and reactive T cells. Moreover, complement system functions as an effector on the clearance of soluble immune complexes and cell debris, which otherwise could induce an immune response against auto antigens and potentially trigger autoimmunity (1–3). Deficiency or dysfunction of the complement system could cause infections in adult patients (4) and also predispose individuals to autoimmune diseases, such as rheumatoid arthritis (RA), systemic sclerosis, and systemic lupus erythematosus (SLE) (5).

**Figure 1:**
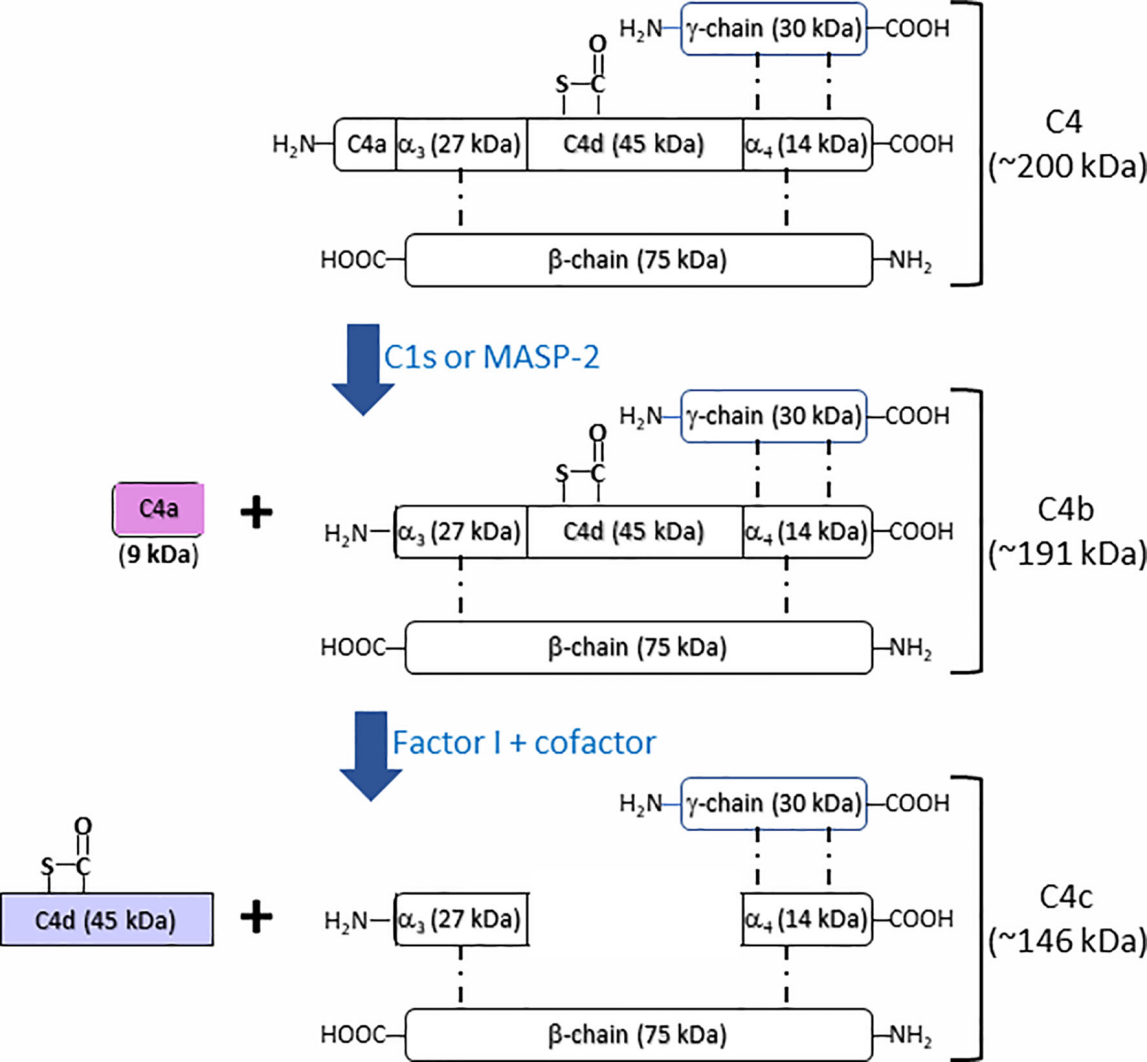
Schematic illustration of fragmentation of complement C4 activation. Complement C4 (~200 kDa) is activated by the serine protease C1s or MASP2 from classical and lectin complement pathway, respectively, to form the activation fragments C4a (~9 kDa) and C4b (~191 kDa). C4b (~191 kDa) is then inactivated and cleaved by the factor I together with cofactors to generate intermediate product iC4b and then further to generate the thioester linked C4d (~45 kDa) and C4c (~146 kDa).

Complement component C4 (Mw = ~200 kDa), an essential component in complement system, plays an indispensable role in the activation of classical and lectin complement cascades. It is a disulfide-bonded three-chain glycoprotein, consisting of an α-chain (95 kDa), a β-chain (75 kDa), and a γ-chain (30 kDa) (**Figure 1**) (6, 7). In the process of the activation of classical complement pathway, C1q from C1 complex [C1q-(C1r)_2_-(C1s)_2_] recognizes antigen–antibody immune complexes or certain membrane-bound structures, *e.g.* C-reactive protein (CRP) or lipopolysaccharides (LPS), resulting in the transition from C1s zymogen to an active C1s repositions, which would be able to interact with sulfotyrosine residues on C4 (8, 9). Similar to the activation of classical complement pathway, the lectin complement pathway is activated by complex carbohydrate structures and mediated *via* recognition molecules as mannan binding lectin (MBL), ficolins, and collectin 10/11, leading to the activation of mannan-associated serine protease-2 (MASP-2), which relies heavily on its active sites, two complement control protein (CCP) domains, and the serine protease (SP) domain for the efficient binding and cleavage of C4 (10–12). As shown in **Figure 1**, the activated C1s and MASP-2 from classical and lectin pathways respectively cleaves the amino terminal part of the α-chain at a single site of complement C4 to generate C4a fragment peptide (9 kDa) and C4b (195 kDa) (13). C4b binds to target surface *via* its reactive thioester, which can be inactivated to an intermediate form iC4b by proteolytic cleavage by the serine protease factor I together with co-factor CD46 (14, 15). iC4b is further cleaved to thioester linked C4d (45 kDa) and soluble C4c (146 kDa), which can be used as a biomarker for complement activation from classical and lectin pathways. Both classical and lectin pathways lead to further activation of C2 to generate C3 convertase C4b2a, which will activate C3 to generate C3a and C3b. C3 convertase binds to C3b to form C5 convertase that will cleave C5 to generate C5a and C5b. C5b binds to C6, C7, C8, and C9 to form membrane attack complexes (MAC) C5b-9 that are formed on the surface of pathogen cell membranes. Comparing crystal and solution structures of C4b with its paralog C3b, their conformations are shown surprisingly conserved (16). Further study revealed that the C3 convertases (C4b2a *vs.* C3bBb) from the classical/lectin and alternative pathways are also strikingly similar, which is in agreement with their identical functions in the cleavage of the downstream complement proteins C3 and C5 (17).

The complete or partial deficiency of complement C4 results in the increased risk of infection and autoimmune diseases. A plethora of studies demonstrated that complement C4 plays an essential role in defensing microbial infection. It is also well established that the complete or partial deficiency of complement C4 is associated with the increased susceptibility to infections (18–23). In addition, the deficiency of complement C4 could lead to various autoimmune diseases (24–33). The reduced concentrations of C4 protein and the reduced serum complement activity occur with the active disease in SLE (25, 34), as well as in infections (35).

In this review, we will look into the updated studies on the role of complement C4 in infectious diseases and autoimmune disorders. In this way, we aim to elucidate and update the functions of complement C4 in infectious diseases and autoimmune disorders, trying to highlight the important role of complement C4 as a potential intervention target for the management of those disorders.

## Diversity of Complement C4 Genes and Proteins

The human complement C4 gene (*C4A* and *C4B* genes) locus is located in the highly polymorphic major histocompatibility complex (MHC) class III gene region on chromosome 6, which could be a short form (C4S, 14.6 kb) or a long form (C4L, 21 kb), depending on the absence or the presence of the 6.36 kb endogenous retroviral sequence HERV-K(C4) in intron 9 of human C4 genes. Three quarters of C4 genes harbor the 6.36-kb endogenous retrovirus HERV-K (C4) (36). Each human C4 gene has 41 exons, which codes for a 5.4 kb transcript. C4 gene lies within a unit of four consecutive genes known as an RCCX module, which stands for the serine/threonine nuclear protein kinase **R**P, **C**omplement component C4, steroid 21-hydroxylase **C**YP21, and extracellular matrix protein tenascin TN**X** (*RP-C4-CYP21-TNX*) (*RCCX*) (37–44). An elevated level of genomic copy number variations (CNV) was shown in MHC region III, supposedly to present immunologic diversity (45). The duplication of these four genes occurs as a module in the class III region of a haplotype for the MHC. The gene copy number (GCN) of *C4A* genes varies from 0 to 5 and GCN of *C4B* genes varies from 0 to 4. The highest total C4 gene dosage reported is 7 (46). It took a long time for scientists to make it clear on the genetic diversity of human complement C4. The initial model was proposed as a single locus of codominant alleles for C4A and C4B, and later two-locus or C4A-C4B models dominated the complement field for about two decades. Extensive molecular and genetic studies have now provided a clear definition of genetic structures that are responsible for C4 isotypes (C4A and C4B) protein expression. Complement C4 protein exists as two isotypes, C4A and C4B, which are encoded by the C4 genes (*C4A* or *C4B* gene*)*, and share >99% sequence identities. Five nucleotide variations located in exon 26 confer to four isotype-specific amino acid substitutions at positions 1101–1106 (**PC**PV**LD** for C4A and **LS**PV**IH** for C4B) and the major structural and functional differences between the C4A and C4B isotypes (47). C4A is named after its acidity and migrates faster in agarose gel electrophoresis as compared to C4B that is basic and migrates slower (47–51). In addition, C4A and C4B are highly polymorphic with more than 40 different alleles, gene duplications, and “null alleles” (52–55). C4A is generally associated with the Rodgers (Rg) blood group antigens and is more reactive with immune complex or the targets containing free amino groups, whereas C4B is generally associated with the Chido (Ch) blood group antigens and is more affinity to hydroxyl groups (56). It was revealed that C4A has a longer half-life in plasma as compared to C4B, suggesting a role of C4A in the clearance of the immune complex and a role of C4B for membrane attack complex formation and the defense against bacterial pathogens (57). The individuals with long C4 genes (C4L) have lower serum levels of complement C4 as compared with short C4 genes (C4S) (36). C4 gene copy number variations (CNV) are correlated to the serum levels of complement C4 protein and low C4 GCNs predisposes individuals with various disease susceptibility (58). Low copy numbers or the deficiency of C4 genes was reported to be one of the strongest risk factors associated with several immune disorders, such as SLE, chronic central serous chorioretinopathy, Behçet’s disease, and Vogt-koyanagi-Harada disease (25, 58–63). It was also reported that the deficiency of either C4A or C4B has been associated with the increased susceptibility of infections (18, 64, 65). Interestingly, it was reported by Bay et al. that low C4 gene copy numbers (< 4 total copies of C4 genes) are associated with superior graft survival in patients transplanted with a deceased donor kidney (66). A comprehensive review on the variations of C4 genetic structures and proteins was presented by Blanchong CA et al. (36).

Other causes than genetic variations also can affect the expression or the function of complement C4. Early studies by Goldman et al. in cultured guinea pig peritoneal cells demonstrated that complement component can be regulated by short-term treatment *in vivo* or *in vitro* with monospecific antibody to individual complement components can have long-term effects on the production of those components. Antibody treatment induced specific suppression of C4 in peritoneal cell monolayers. Further studies revealed that long-term C4 suppression is actively maintained by a soluble suppressor factor (FsC4) (67–70). Most of those experiments were carried out *in vitro* cellular models from guinea pig. It is still unclear whether this observation can be replicated in human.

Dysregulation of classic and lectin complement pathways that complement C4 participates in can lead to complement-mediated autoimmune diseases. C4 nephritic factor (C4Nef), first described by Halbwachs et al. in 1980, is an autoantibody to C3 convertase (C4b2a). C4Nef can prolong the half-life of C3 convertase by stabilizing C4b2a and protects C4b2a against decay dissociation by C4 binding protein (C4BP). Multiple clinical studies demonstrated that C4NeF was associated with post-infectious acute glomerulonephritis, systemic lupus erythematosus, chronic proliferative glomerulonephritis and hypocomplementemic membranoproliferative glomerulonephritis (MPGN), and meningococcal disease (71–76).

A recent study by Battin et al. using *in vitro* binding screening demonstrated that Neuropilin-1 (NRP1) acts as a receptor for complement split products, such as C4d, C3d, and iC3b. NRP1 is a highly conserved type 1 transmembrane protein that is involved in the tumorigenesis, the development of cardiovascular, and nervous systems through the interaction with vascular endothelial growth factor (VEGF) and semaphoring 3A (Sema3A). NRP1 is also expressed in murine immune cells and serves as a marker for mouse T_reg_ cells. Interestingly, NRP1 was demonstrated to bind C4d in a concentration-dependent and saturable manner. These data demonstrated NRP1 functions as a receptor for C4d that is covalently bound to target surfaces during complement activation, suggesting that NRP1 might be involved in regulation of the process of infections and autoimmune disorders by targeting the split product from classical or lectin complement pathway (77).

## Regulation of the Activation of Complement C4

### Complement C4 in Microbial Infection

Complement C4 is involved in the activation of both classical and lectin complement pathways. The classical pathway of complement system is crucial for anti-microbial defense through anti-pathogen antibody, which recruits C1 complex and initiates a cleavage cascade involving C4, C2, C3, and C5 and accomplishing microbial clearance. In addition, lectin complement pathway is also involved in the anti-microbial defense. Recent study revealed that loss of classical pathway results in rapidly progressing septicemia and impaired macrophage activation, suggesting that the classical pathway is the dominant pathway for activation of the complement system during complement innate immunity to *S. pneumoniae*. In response to microbial pathogens, lectin pathway is activated as an innate immune response through direct binding to bacterial surface sugar components. In contrast, the classical pathway was an effector of adaptive immune response through activation of antibody–antigen complexes on bacterial surfaces and plays a vital role partially targeted by the binding of natural IgM to bacteria (21).

An early research work by Schifferli et al. demonstrated that C4A isoform of complement C4 was more efficient than C4B isoform in the processing of immune complexes in humans. In contrast, hemolysis by C4B isoform was more efficient than by C4A isoform, suggesting that both C4 isoforms are complementary (78). A recent study by Liesmaa et al. demonstrated that homozygous C4A deficiency in patients was associated with the increased prevalence of lymphomas, celiac disease, and autoimmune disease SLE. In the same study, homozygous C4B deficiency in patients was documented to be linked with the drug intolerance and various post-infectious symptoms. Homozygous C4B deficiency alone is not considered as a significant factor in causing invasive infection (79). From the multiple studies from different laboratories, it seems still debatable in terms of the role of homozygous C4A or C4B deficiency in infection-proneness of an individual (64, 79–82).

Complement interfering protein (CIP) expressed on the surface of group *B. Streptococcus* (GBS) enables cell adhesion and penetration and impedes innate and adaptive immune responses. It was found that CIP was able to interact with the human C4b ligand and to interfere with the classical- and lectin-complement pathways (83). Clinical *Staphylococcus aureus* (*S. aureus*) strains can recruit complement regulator C4-binding protein (C4BP) to *S. aureus* surface to inhibit C4 complement effectors through binding significant amounts of the C4BP from serum. The complex (*S. aureus*-bound C4BP) functions as a cofactor for factor I-mediated C4b cleavage to iC4b and C4d, which was used as a strategy by *S. aureus* for immune evasion (84).

A recent study revealed that gram-negative Bordetella pertussis could evade the attack from the human complement system by releasing virulent protein Vag8 of *B. pertussis*. Endogenously secreted and recombinant Vag8 can inhibit complement deposition on the bacterial surface at the level of C4b. The binding of C1 inhibitor (C1-inh) to C1s, C1r, and MASP-2 was disrupted by the association of Vag8 with human C1-inh, which will free active proteases to cleave C2 and C4 away from the bacterial surface, revealing a mechanism of the unique complement evasion strategy of *B. pertussis* (85).

An alkaline protease Alp1p secreted from *A. fumigatus mycelia* can facilitate early immune evasion by deactivating the complement defense in the human host, either by directly cleaving the complement components C3, C4, and C5 or by cleaving them to a form that is further fragmented by other proteases (86, 87).

Some small organisms other than microbials are also involved in the activation or the inhibition of complement systems. For examples, complement activation-inhibition substance from maggot excretions, which splits complement proteins C3 and C4 in a cation-independent manner, could provide a novel treatment for several diseases that result from the activation of complement system (88). ES-62, a protein with an N-linked glycan linked with phosphorylcholine (PCh) produced from parasitic nematodes, was bound to C-reactive protein (CRP) in normal human serum. C1q can capture ES-62-CRP to form a larger complex ES-62-CRP-C1q in serum. Following CRP interaction, ES-62 was able to deplete early complement component C4 and inhibit classical pathway activation (89). The immune evasion strategies used by those microorganisms aforementioned were summarized in **Table 1**.

**Table 1:** The evasion mechanisms of microorganisms from the attack of innate immunity of complement system by targeting complement C4.

Microorganisms	Molecule(s) involved	Expression or Function of C4	Reference
Hepatitis C			
HCV Core protein	USF-1 and C4 mRNA	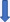	(90)
HCV NSSA	IRF-1 and C4 nRNA	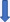	(90)
HCV NS3/4A	Cleavage of C4 γ-chain	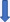	(91)
HCV NS2/NS5B	Disruption of the interaction of MICA/B and NKG2D	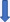	(92)
Flavivirus (DENV, WNV, YFV)			
NS1	Cleavage of C4	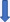	(93)
*B. Streptococus*	Complement interfering protein (CIP)	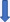	(83)
*S. aureus*	C4-binding protein (C4BP)	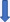	(84)
*B. pertussis*	Vag8	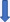	(85)
*A. fumigatus mycelia*	Alkaline protease Alp1p	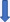	(86, 87)
Maggot	Inhibition substance of complement activation	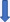	(88)
Parasitic nematodes	ES-62	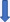	(89)

USF-1, upstream stimulating factor 1; IRF-1, interferon regulatory factor 1; MICA/B, major histocompatibility complex class I-related chains A and B; NKG2D, a transmembrane protein belonging to the NKG2 family of C-type lectin-like receptors.

It is now becoming apparent that microbial organisms generate various mechanisms to defend the attacks from innate immunity of complement system. Elucidation of those mechanisms will potentially provide strategies to treat microbial infectious diseases, as well as to explore the anti-complement therapeutic interventions.

### Complement C4 in Viral Infection

#### Infection of Hepatitis B

There are extensive studies on the role of complement system in viral infections (93–95). A study by Bugdaci et al. was carried out in 143 patients on the relationship of serum complement C4 levels and chronic hepatitis B (CHB) infection with high transaminase level. Serum C4 levels in patients with CHB with high transaminase level were found significantly lower. In addition, Child score in patients with cirrhosis inversely correlated with C4 levels, suggesting that the levels of complement C4 in plasma significantly correlate with liver biopsy findings and may be a useful indicator of disease activity and/or damage in patients with CHB with high transaminase levels (96).

#### Infection of Hepatitis C

A study by the same research group of Bugdaci et al. on 100 patients with chronic hepatitis C found that complement C4 levels showed significant correlation with alanine aminotransferase but could not find any relationship between serum complement C4 level and fibrosis (97). It remains unanswered why C4 activity was significantly lower in patients with chronic hepatitis C virus (HCV) infections. One speculation could be due to excessive activation of C4 protein by the activation of classical and lectin complement pathways during HCV infections. Several studies evaluated the expression of C4 in terms of anti-HCV therapeutic response and disease progression in chronic hepatitis C (CHC) patients. The studies revealed that mRNA and protein levels of complement C4 were significantly increased after anti-HCV treatment. A positive alteration in C4 level represents as an independent predictor for treatment response and reflects viral clearance after anti-HCV therapy in CHC patients (98–100). Further studies revealed that hepatitis C virus proteins [HCV core; non-structural (NS) 5A] render transcriptional suppression of the expression of complement C4. Liver biopsy specimens from chronic HCV patients displayed significantly lower levels of complement C4 mRNA as compared to the liver tissue samples from patients with other types of liver disease. HCV core protein was found to decrease the expression of upstream stimulating factor 1 (USF-1), a transcription factor essential for basal C4 expression. In addition, HCV NS5A protein can inhibit the expression of interferon regulatory factor 1 (IRF-1), which is important for IFNγ-induced complement C4 expression. These results highlight the roles of HCV proteins in establishing a chronic infection through the regulation of innate immunity by affecting the expression of complement C4 (90).

Another study by Mawatari et al. demonstrated that HCV NS3/4A protease could cleave the γ-chain of complement C4 in a concentration-dependent manner, suggesting that complement C4 is a novel cellular substrate of HCV N3/4A protease, which reveals new insight into the mechanisms underlying persistent HCV infection (91).

Natural killer (NK) cells have been revealed to contribute to regulating complement synthesis. Studies using co-culture of NK cells (NK3.3) with human hepatoma cells (Huh7.5) expressing HCV core or NS5A protein revealed a significantly increased synthesis of complement C4 and C3 *via* increased specific transcription factors. The regulatory activity is mediated through a direct interaction between the hepatocyte protein major histocompatibility complex class I-related chains A and B (MICA/B) and NKG2D on NK cells. However, when NK cells were co-cultured with Huh7.5 cells infected with cell culture-grown HCV, complement C4 and C3 synthesis was impaired. MICA/B expression in HCV-infected hepatocytes was found to be repressed during co-culture because the HCV-associated expressions of NS2 and NS5B proteins can disable a crucial receptor ligand in infected hepatoma cells, resulting in the disability of infected cells to respond to stimuli from NK cells to up-regulate the expression of complement C3 and C4 (92). This piece of data revealed that HCV synthesizes the proteins that can down-regulate complement C4 expression to evade the attack from complement systems.

#### Infection With Flaviviridae and Other Viruses

Flavivirus infection, such as West Nile virus (WNV) and Dengue virus (DENV), was restricted through an antibody-independent fashion. N-linked glycans on the structural proteins of flaviviruses was recognized by mannose-binding lectin (MBL), resulting in neutralization and efficient clearance *via* a C3- and C4-dependent mechanism that applied both the canonical and bypass complement lectin activation pathways, which recognizes terminal mannose-containing carbohydrates on the viruses (101). A recent study by Avirutnan et al. demonstrated that flavivirus non-structural (NS)1 protein from dengue virus (DENV), West Nile virus (WNV), and yellow fever virus (YFV) binds to C4 to enhance cleavage of C4 and reduce C4b deposition and C3 convertase (C4bC2a) activity that confers to immune evasion function for the viruses (93).

Interestingly, Puumala (PUUV) hantavirus triggers complement system activation *via* the alternative pathway, which is complement C4-independent, causing the increase of sC5b-9 and the decrease of C3. In the acute stage of PUUV infection, the level of complement activation correlates with disease severity, indicating that complement activation may contribute to the pathogenesis of acute PUUV infection (102).

A recent study by Bottermann et al. revealed a novel antiviral mechanism that is C4-dependent and late-acting complement components-independent. C4 inhibits human adenovirus infection by the deposition of cleaved C4b on capsid, which inhibits it disassembly, preventing endosomal escape and cytosolic access (103). The mechanisms that viruses applied to downregulate the expression or the function of complement C4 are summarized in **Table 1**.

#### Coronavirus (SARS-CoV1, SARS-CoV-2, and MERS-CoV)

In the midst of pandemic with severe acute respiratory syndrome coronavirus 2 (SARS-CoV-2)/COVID-19. The infection involves in multiple organs and cause striking elevations in pro-inflammatory cytokines and high risk of thrombosis. Numerous postmortem studies have revealed deposits of complement fragments on interalveolar endothelial cells, high incidence of venous thromboembolism (VTE), and diffused microvascular thrombi with endothelial swelling with a thrombotic microangiopathy (TMA). Preclinical studies with SARS-CoV-1 and MERS-CoV, which have significant homology to SARS-CoV-2, confirm that complement activation is not only linked to virus related organ damage but also is possible causative (104). In mouse models with infection with MERS-CoV or SARS-CoV-1, increased tissue deposition of C5b-9, C3b, and C4d and correlation with severity of injury were observed. Given the fact that deficiency of complement C3, C4, and factor B can protect mice from virus caused by lung injury, classical, lectin, and alternative complement pathways might be involved in mediating SARS-CoV-1 or SARS-CoV–triggered lung injury (105, 106). In the few published post-mortem studies of COVID-19 patients, the increased deposits of C3b, MBL, MASP-2, C4b, and C5b-9 were observed (107, 108). It shows excessive activation of lectin pathway, which is in line with the fact that the spike protein in SARS-CoV-2 is heavily glycosylated with l-fucose and mannose, which provides recognition sites for MLB binding and causes activation of lectin pathway (109). There is no doubt that complement C4 will be hyper-activated in SARS-CoV-2 infection. Is there any relationship between the complement C4 activation and COVID-19 infection caused endothelial swelling and diffused microvascular thrombi that resemble TMA? We would speculate that the hyperactivation of lectin pathway might cause endothelial disruption that might be one of the mechanisms to induce microvascular thrombi in COVID-19 patients. It remains to be evaluated how the lectin pathway and complement C4 activations cause endothelial swelling and diffused microvascular thrombi.

### Complement C4 Activation Under Other Pathological Conditions

A recent study by Romano et al. revealed that anti–interleukin-6 receptor monoclonal antibody (Tocilizumab) could dramatically decrease serum level of complement C4 in rheumatoid arthritis patients. Neither circulating immunocomplexes nor any patients ever displaying clinical features of immunocomplex diseases was found. The study concluded that C4 consumption is because of the direct action of the drug rather than immunocomplex-induced complement activation (110).

Histone H3 and H4 can be released from the damaged or lysed cells. One recent study by Qaddoori et al. revealed that histone H3 and H4 strongly bind to C4b region of complement C4, result in significant inhibition of classical and mannose-binding lectin pathways. Histone H3 and H4 did not affect the cleavage of C4 to C4a and C4b, indicating a possible natural feedback mechanism to prevent the excessive injury of host cells by the inhibition of complement activation by histones (111).

**Figure 2:**
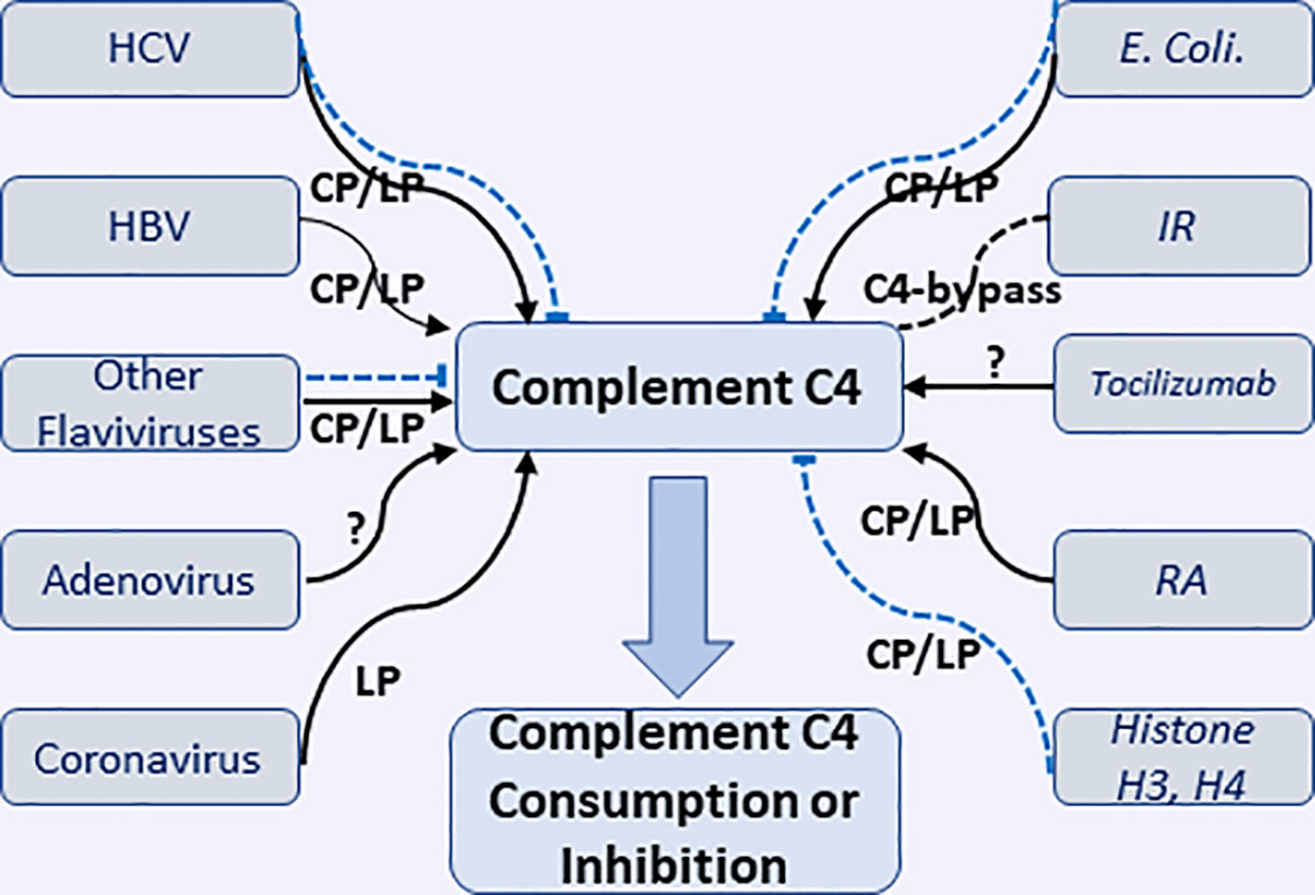
Interactions of various microorganisms (including viruses and *E. coli.)* and pathological conditions with complement C4 to cause consumption or inhibition of complement C4 through classical, lectin, and undefined pathways. HCV, hepatitis V virus; HBV, hepatitis B virus; IR, ischemia reperfusion; RA, rheumatoid arthritis. Black solid arrow represents the activation of complement C4; Blue dash line represents the inhibition of complement C4.

A recent study by Vogt et al., using highly specific antibody against a cleavage neoepitope in C4d, identified pigment epithelium-derived factor (PEDF) from synovial fluid of rheumatoid arthritis patients as an activator of classical complement pathway, which belongs to the serine proteinase inhibitor family. C1q protein can bind PEDF, in particular, head regions of C1q, which is known to interact with other activators of the classical pathway. The results suggested PEDF activated classical complement and might mediate inflammatory processes in joint (112). The interactions of virus, bacteria, and some pathological conditions causing the consumption or inhibition of complement C4 through classic or lectin complement pathway are illustrated in **Figure 2**.

Using computational approach (protein-protein docking and molecular dynamics simulation), a recent study tried to understand Trypsin (Tryp)-mediated C4 activation by comparing with the co-crystalized structure of C4-MASP2. Comparative analysis of C4 alone, C4-Tryp, and C4-MASP2 discovered the impact of Tryp on C4 was like that of MASP2. These studies define the role of sessile loop in the interaction with serine domain, which could be beneficial to understand the interactions of complement C4 with other complement components (113).

### C2- and C4-Bypass Lectin Pathways

It seems that sometimes the activation of three complement pathways is not clear-cut. Recent studies in mice established that the complement activation *via* the alternative pathway requires the presence of C4 and MBL proteins and the complement activation by *Cryptococcus* spp. can take place *via* multiple complement pathways (114, 115).

Although complement C4 does not directly participate in the activation of alternative complement pathway (AP), several early studies from the 1980s to 1990s of last century demonstrated that C4b generated from classical pathway activation could trigger the alternative pathway without involvement of complement C2 (116–118). It was reported that MBL can activate complement C3 and AP without the involvement of MASP-2, C2, or C4 (119, 120). A recent study by Tateishi and Matsushita demonstrated that upon the attachment to serogroup C-specific oligosaccharide from *Salmonella*, in contrast to that MBL activates the alternative pathway *via* a C2-bypass pathway without the involvement of MASP-2, C2 or C4, mannan-bound MBL can activate the alternative pathway *via* a C2-bypass pathway that requires both MASP-2 and C4, suggesting that there may be two distinct MBL-mediated C2-bypass activation of alternative complement pathway, depending on the ligands to which MBL binds (121). It seems that there are some issues related to those *in vitro* assays. First, those MBL preparations could possibly have the trace contamination with MASP-2. Another question is how pure the serum preparations with the depleted MBL, C2, or C4 can be. The mechanism of MLB mediated C2-bypass AP activation remains to be determined and further studies are needed to elucidate the molecular base of MBL-mediated C2-bypass pathways as indicated in the paper.

**Figure 3:**
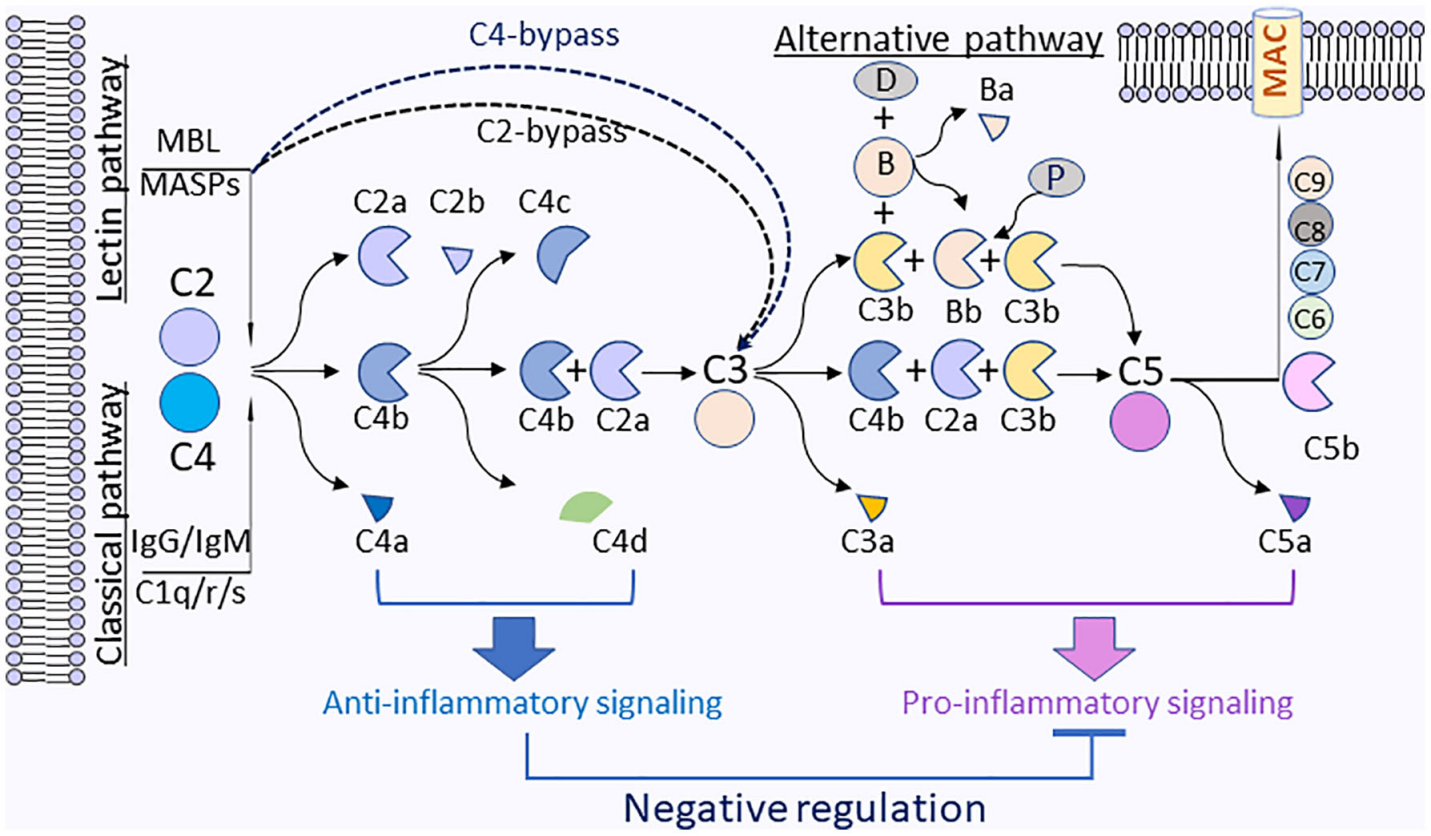
Anti-inflammatory functions by complement C4 activation fragments C4a and C4d. On the surface of pathogens, the activation of complement C4 is triggered through classical (antibody) or lectin (sugar) pathways that will activate C1s or MAPSs, which will rapidly cleave C4 to generate C4a and C4b. C4b will be further fragmented by factor I and cofactors to generate C4d and C4c. C4b will associate with C2a to form a complement C3 cleavage enzyme (C3 convertase), C4b2a, which will cleave C3 to generate C3a and C3b. C3b will be associated with C4b2a to converge to C5 convertase (C4bC2aC3b), which will cleave C5 to generate C5a and C5b. C5b will associate with C6, C7, C8, and C9 to form membrane attack complexes (MAC) C5b-9 on the surface of pathogens. For the alternative pathway, C3b is spontaneous C3 turnover or generated by classical or lectin pathways. C3b bound to factor B (B). The complex is converted by factor D (D) to C3-cleaving enzyme C3bBb that is stabilized by properdin (P) and further form C3bBbC3b, which can cleave C5 to generate C5a and C5b. Plasma membrane (blue) represents the surface of pathogen cells.

Several studies suggested that MLB complement pathway could activate C3 or C5 through C4-bypass mechanisms (119, 122–125). The study in a mouse model by Schwaeble et al. demonstrated that in the absence of complement C4, *in vitro* lectin pathway-mediated activation of C3 requires MASP-2, C2, and MASP-1/3. In a model of transient myocardial postischemic reperfusion injury (IRI), comparing to their wild-type littermates, infarct volumes of MASP-2-deficient mice were smaller. However, mice deficient in complement C4 were not protected, the observation implies the presence of a previously undocumented C4-bypass and lectin pathway-dependent mechanism. As monoclonal Antibody-based inhibitors of MASP-2 and MASP-2 deficiency can also protect mice from gastrointestinal IRI, suggesting the benefit of anti-MASP-2 antibody therapy in reperfusion injury and other lectin pathway-mediated disorders (126). In this study, it was unclear how the correlation between complement C3 activation by C4-bypass lectin pathway and the disease state of infarct volume. IRI may not be due to the complement C3 activation, but could be attributed by MASP-2- or MASP-1-induced direct activation of coagulation systems, leading to the formation of a fibrin clot (127, 128). Lectin pathway complement activation plays a critical role in contributing to ischemia reperfusion (IR) injury. One recent study in a mouse model corroborates the effect of MASP-2, an essential enzyme for lectin pathway, which mediates tissue injury and renal ischemia reperfusion injury independent of complement C4 (129). C2- and C4-bypass lectin pathways activation are depicted in **Figure 3**.

Although classical and the alternative pathways can still be activated, MASP-2 deficient mice fail to opsonize *Streptococcus pneumoniae* in the none-immune host and therefore are highly susceptible to pneumococcal infection. Mouse ficolin A, human L-ficolin, and collectin 11 in both species, but not mannan-binding lectin (MBL), are the pattern recognition molecules that drive lectin pathway activation on the surface of *S. pneumoniae*. pneumococcal opsonization in the absence of complement C4. This study corroborates the crucial function of MASP-2 in the lectin pathway and underlines the prominence of MBL-independent lectin pathway activation in the host defense against pneumococci (130).

Recent study in mice demonstrated that MASP-2 deposits complement C4 onto mitochondria, revealing the potential role of the complement lectin pathway in mitochondrial immune handling. These processes are speculated to be involved either in the induction of problematic inflammatory reactions or in homeostatic clearance of mitochondria (131).

As discussed above, the complement lectin pathway has a protective function against invading pathogens and plays an essential role in ischemia/reperfusion (I/R)-injury as well. The serpin C1-inhibitor and aprotinin, a Kunitz-type inhibitor can inhibit MASP-2. Recombinant tissue factor pathway inhibitor (rTFPI) was identified as a novel selective inhibitor of MASP-2, without disturbing the activity of MASP-1 or the classical pathway proteases C1s and C1r. Ex vivo assay revealed that Kunitz-2 domain in TFPI was necessary for the inhibition of MASP-2 activity. TFPI could be a therapeutic approach to constraint the tissue injury in the conditions of cerebral stroke, myocardial infarction, or solid organ transplantation (132).

## The Signaling Pathways of C4 Activation Fragments

C4a, one of the activation fragments of complement C4, identified in 1979, was regarded as the third anaphylatoxin although it is still under debate (133, 134). C4a has been described to possess a strong chemotaxis inhibitory effect on monocytes at the concentration as low as 10^-16^ M (135). C4a was also reported to inhibit C3a-induced O^2·−^ generation in guinea pig macrophages, to produce immediate erythema/edema when injected into human skin, and to induce contraction of guinea pig ileum (133, 136). It was suggested that a function for C4a is closely related to C3a due to its ability to desensitize the action of C3a-induced contraction of guinea pig ileum (133). It was later revealed that human C4a acted as an agonist for the guinea pig but not the human C3aR receptor (137). Studies using recombinant human C4a have also demonstrated that C4a can impair C5a-induced neointima formation, reduce C3a- or C5a-mediated chemoattractant and secretagogue activities in mast cells, and prevent hyperoxic lung injury *via* a macrophage-dependent signaling pathway (138–140). It remains to be established how C4a can modulate the functions of monocytes/macrophages to generate anti-inflammatory effects.

C4d, another cleavage product by complement C4 activation, has long been considered as a biomarker for disease activity in autoimmune disorders or antibody-mediated allograft rejection. A recent study identified Ig-like transcript (ILT) 4 and ITL5v2 as cellular receptors for C4d and interaction of C4d with ILT4 conferred a dose-dependent inhibition of TNFα and IL-6 secretion and attenuation of intracellular [Ca^2+^] flux in monocytes activated *via* Fc-cross-linking of up to 50% as compared to control (141). ILT4 has been involved in the control of autoreactivity (142, 143), induction of transplantation tolerance (144), and maintenance of feto-maternal tolerance during pregnancy (145). Mice lack of PIR-B, the ortholog of ILT4, suffer from autoimmune glomerulonephritis (146) and exacerbated graft versus host disease (147). It appears that the interactions of complement C4 activation cleavage fragments (such as C4a and C4d) with their respective receptors plays inhibitory roles to impede inflammatory reactions induced by cytokines, chemokines, and other anaphylatoxins (C3a, C5a). One interesting paradigm will be that upon complement activation (*i.e.*, microbial or viral infection, immunocomplex, or apoptotic debris), complement C4 activation fragments may act as regulators to maintain homeostasis and to contain downstream anaphylatoxins’ proinflammatory effects, which may trigger hyper-inflammatory reactions. The activation of complement C4 and potential immune-regulation mechanisms of split products from complement C4 are illustrated in **Figure 3**.

## Complement C4 Deficiency Links Infections and Autoimmune Diseases

It remains incompletely understood why total deficiencies of some complement components are associated with some autoimmune diseases (148). Complement C4 plays a vital role in the activation of classical and lectin pathways and the formation of C3 convertase, which leads to the generation of the membrane attack complex (MAC) against microbes. It was reported that complement C4 is protective for autoimmune lupus disease independent of C3 in mice (30). C4(-/-) mice have significantly more IgM anti-double-strand DNA antibodies than C4(+/+) control mice (32). Increased frequency of C4 deficiency phenotypes was reported in IgA nephropathy and Henoch-Schönlein purpura (HSP) (26), insulin-dependent diabetes mellitus (IDDM) (149), systemic lupus erythematosus (SLE) (150, 151), repeated infections (152), juvenile idiopathic arthritis patients (27). glomerulonephritis (153). The lack of complement C4 can trigger inapt clearance of apoptotic debris and stimulate chronic activation of myeloid cells. The deficiency in complement component C4 also results in a breakdown in the elimination of autoreactive B-cell clones at the transitional stage, depicted by a relative increase in their response to a series of stimuli, entering into follicles, and a higher tendency to form self-reactive germinal centers (GCs), allowing the maturation and activation of self-reactive B-cell clones (154). Using two well-defined murine models to examine complement deficiency in peripheral tolerance, the study revealed that complement C4 protein and the receptors CD21/CD35 are involved in negative selection of self-reactive B lymphocytes, suggesting an immune deficiency of complement C4 predisposes mice to SLE (29). A low serum C4 level in patients with autoimmune disease may be due to ongoing disease activity associated with the consumption caused by complement activation and or it may be due to genetic factors (155). One of the questions still remains: whether and how does infections link to autoimmune disease upon the deficiency of complement C4? It is speculated that C4 deficiency would negatively affect the efficiency and progression of complement activation, decrease phagocytes functions and the clearance of apoptotic and necrotic cells (156).

A recent study by Yammani et al. demonstrated that complement C4 deficiency is a predisposing factor for streptococcus pneumonia-induced anti-dsDNA IgA autoantibody production. In a C4KO mice model, serotype 19F and virulent serotype 3 pneumococci induce systemic anti-dsDNA IgA production; interestingly which is more pronounced in female C4KO mice. Further study revealed that pneumococci pneumococcal polysaccharide (PPS) vaccination alone induced increases in anti-dsDNA IgA levels, which can be completely blocked by TLR2/4 antagonist, OxPAPC. Pam3CSK4, a TLR2 agonist, equally stimulated anti-dsDNA IgA production in C4KO mice, suggesting complement C4 plays a role in subduing autoantibody production stimulated by cross-reactive antigens and TLR2 agonists associated with *S. pneumonia* (33).

A complete analysis of the potential Epstein-Barr virus (EBV) peptide cross-reactome has been performed to search for peptides common to SLE-EBV and human SLE autoantigens. The study found EBV proteome can act as an immunological potential. Using publicly available databases, fifty-one SLE-related proteins were analyzed for hexapeptide sharing with EBV proteome and found 34 of hexapeptides are shared between human SLE autoantigens and EBV proteins. Interestingly, the study also revealed that peptide sharing mostly occurred with complement component C4 and Interleukin-10 (IL-10). This study demonstrated that the EBV *vs*. SLE autoantigens peptide overlap and powerfully supports cross-reactivity as a major mechanism in EBV-associated SLE etiopathogenesis (157). Among patients with systemic lupus erythematosus (SLE), a prevalence of HPV infection has been reported. One interesting hypothesis is that immune responses caused by HPV infection may interact with proteins that associate with SLE (158).

In lymphoid tissues and peripheral blood of C4KO mice, it was discovered with the decreased frequencies of CD4^+^CD25^+^ T_regs_ and reduced expression of Foxp3 and TGF-β, which are crucial for the efficient development and function of T_regs_ cells. Thus, the study suggested that the association of the deficiency of complement C4 in the classical complement pathway with the development of autoimmune disorder might be *via* the role of T_regs_ deficiency (159). It remains to be elucidated how the fragments generated from complement C4 activation, such as C4a and C4d, are regulating T_regs_ cells functions.

## Concluding Remarks

Complement system is essential for the maintenance of homeostasis by elimination of immune complexes, supporting self-tolerance and anti-inflammation, and promoting tissue repair (160, 161). While the complement activation of the downstream of complement C3 resulting in inflammatory molecules, such as C3a, C5a, and the membrane attack complex (MAC), plays a less important role, the early components of the classical pathway, such as C1q, C4, and C2, are more critical in maintaining homeostasis and lack of some of early components of classical pathway will predispose an individual to autoimmune disorders. Many studies have linked the complement C4 deficiency/partial deficiency with autoimmune disorders. In addition, C4 deficiency is clearly linked to the susceptibility of infections. Those observations persuade us to speculate that infection-caused inflammation needs the containment that requires the immune modulation from the contribution of complement C4, otherwise it will be aggravated under the deficiency of C4. Complement C4 is reported to be chiefly expressed in hepatocytes, but the upregulation of mRNA expression of complement C4 was observed by LPS, IFNγ, and interleukin-6 in other types of cells, indicating that infection-induced cytokines could trigger the upregulation of complement C4 expression as a feedback regulatory response. Mounting evidence supports the observation that infections may initiate and/or exacerbate autoimmune reactions (162–165), which is in line with the studies in mouse models that have established the role of complement C4 as suppressing auto-antibody production (31–33, 166). Nevertheless, the mechanisms of complement C4 involved in homeostasis still have been poorly addressed. Recent studies demonstrated that complement C4 activation fragments, like C4a and C4d, can modulate cytokines generation from macrophages probably through their respective receptors. One of possible mechanisms that complement C4 mediated homeostatic process might be *via* its activation fragments, which can modulate immune reactions to restrain infection-induced hyper-inflammatory reactions induced by cytokines and anaphylatoxin C3a and C5a (**Figure 3**).

Future studies are necessary to focus on the immune regulatory functions of C4 activation fragments, which will be explored as therapeutic targets for the treatment of infections, as well as the autoimmune disorders.

## Author Contributions

HW contributed to the conception and idea of the work. All authors contributed to the article and approved the submitted version.

## Funding

Research work was supported by the seed grant (to HW) from College of Pharmacy and mini-grant (to HW) from College of Medicine, California Northstate University, Elk Grove, CA 95757, United States.

## Conflict of Interest

The authors declare that the research was conducted in the absence of any commercial or financial relationships that could be construed as a potential conflict of interest.
